# Cellular Plasticity and Heterotypic Interactions during Breast Morphogenesis and Cancer Initiation

**DOI:** 10.3390/cancers14215209

**Published:** 2022-10-24

**Authors:** Saevar Ingthorsson, Gunnhildur Asta Traustadottir, Thorarinn Gudjonsson

**Affiliations:** 1 Stem Cell Research Unit, Biomedical Center, School of Health Sciences, University of Iceland, 101 Reykjavik, Iceland; 2 Faculty of nursing and midwifery, School of Health Sciences, University of Iceland, 101 Reykjavik, Iceland; 3 Department of Pathology, Landspitali University Hospital, 101 Reykjavik, Iceland; 4 Department of Laboratory Hematology, Landspitali University Hospital, 101 Reykjavik, Iceland

**Keywords:** mammary gland, breast cancer, plasticity, stem cells, microenvironment, tumor progression, tumor initiation

## Abstract

**Simple Summary:**

This review aims to discuss the structure, function and dynamics of the breast gland and how changes to the function of the breast’s cells can lead to different types of cancer.

**Abstract:**

The human breast gland is a unique organ as most of its development occurs postnatally between menarche and menopause, a period ranging from 30 to 40 years. During this period, the monthly menstruation cycle drives the mammary gland through phases of cell proliferation, differentiation, and apoptosis, facilitated via a closely choreographed interaction between the epithelial cells and the surrounding stroma preparing the gland for pregnancy. If pregnancy occurs, maximal differentiation is reached to prepare for lactation. After lactation, the mammary gland involutes to a pre-pregnant state. These cycles of proliferation, differentiation, and involution necessitate the presence of epithelial stem cells that give rise to progenitor cells which differentiate further into the luminal and myoepithelial lineages that constitute the epithelial compartment and are responsible for the branching structure of the gland. Maintaining homeostasis and the stem cell niche depends strongly on signaling between the stem and progenitor cells and the surrounding stroma. Breast cancer is a slowly progressing disease whose initiation can take decades to progress into an invasive form. Accumulating evidence indicates that stem cells and/or progenitor cells at different stages, rather than terminally differentiated cells are the main cells of origin for most breast cancer subgroups. Stem cells and cancer cells share several similarities such as increased survival and cellular plasticity which is reflected in their ability to switch fate by receiving intrinsic and extrinsic signals. In this review, we discuss the concept of cellular plasticity in normal breast morphogenesis and cancer, and how the stromal environment plays a vital role in cancer initiation and progression.

## 1. Histological Context of the Breast Gland

**Figure 1:**
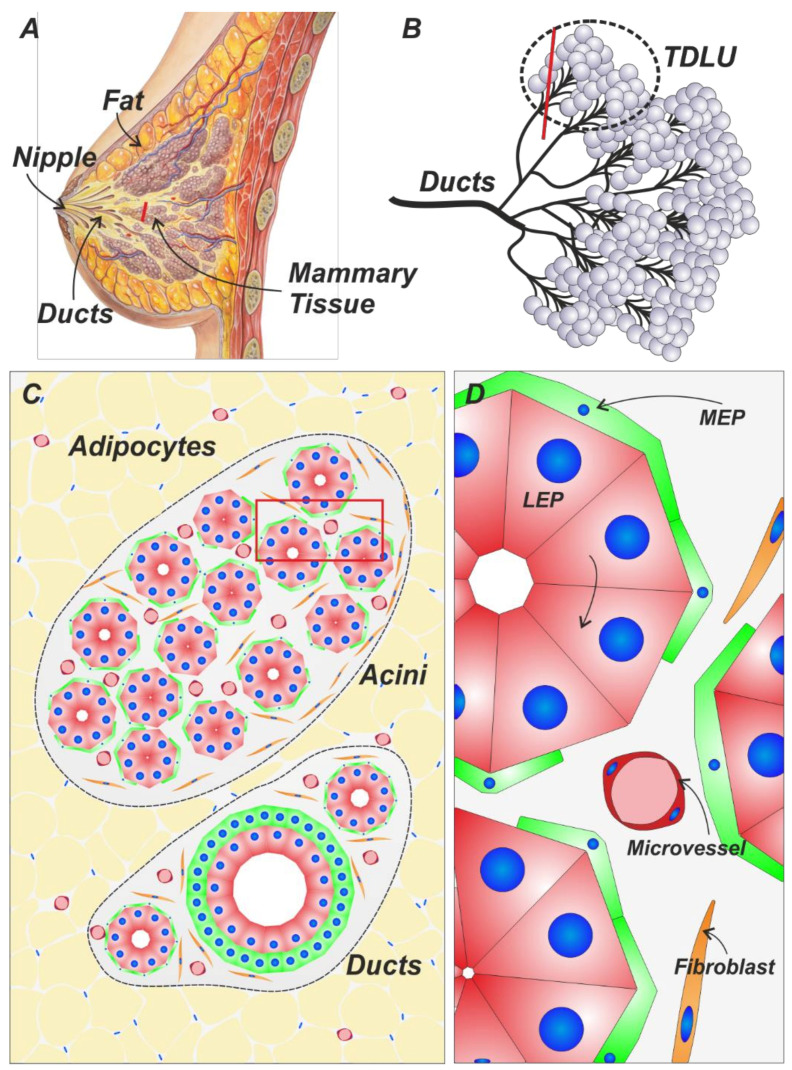
(**A**) The breast gland is composed of branching epithelial ducts that are embedded in collagen and fat-rich stroma. (**B**) These ducts terminate in the TDLU, the functional, milk-producing unit of the breast gland. Artwork in panel A is adapted from Patrick J. Lynch (CC BY 3.0). Red line depicts a virtual cut shown in panel C. (**C**) The TDLU is composed of acini and small ducts, joining together into larger ducts. The mammary tissue is embedded in a collagen-rich stroma, further embedded in a fatty connective tissue. The red box delineates the area magnified in panel D. (**D**) Mammary acini and ducts are arranged in double-layered structures. LEPs are situated towards the lumen, MEPs line the outside. Different cellular components within the breast gland can be characterized using identifying markers.

The human breast gland is a histologically diverse and dynamic organ. In its basic structure, it is a specialized apocrine gland, originating in the nipples as the opening of several ducts. Due to signals from the mesenchyme/stroma, these ducts branch deeper into the underlying connective tissue (Figure 1A,B). The ducts terminate after several branching points in the terminal duct lobular units (TDLUs), which constitute the functional units of the breast gland. In the TDLUs of a lactating breast gland, milk is secreted and ejected upon suckling (Figure 1C). Histologically, the epithelial compartment is in simplest terms organized as a binary layer of epithelial cells. Inside, towards the lumen, the luminal epithelial cells (LEPs) are found, surrounded by a layer of myoepithelial cells (MEPs) that contract as a response to suckling. In the acini of the TDLUs LEPs secrete milk, and MEPs form a basket-like pattern, instead of a continuous thicker layer, as can be seen in the ductal system (Figure 1C,D). The epithelial compartment resides on the basement membrane which is surrounded by collagen-rich stroma that is produced and maintained mainly by fibroblasts. The stroma can be subdivided into two compartments: the intralobular stroma found within the TDLU, and the interlobular stroma, filling the spaces between TDLUs. The intralobular stroma is loose areolar connective tissue, rich in fibroblasts, immune cells, and microvasculature. It has been demonstrated in recent studies to be functionally and developmentally distinct from the interlobular stroma, which is denser and more tightly packed with extracellular matrix proteins such as collagen. To some extent, these two areas are akin to the papillary and reticular connective tissues of the skin, respectively [1].

In the last decade, accumulating evidence has shown that the two different connective tissue areas, commonly referred to as the stroma, are phenotypically and functionally different [2,3]. This may be important for the development and function of the breast gland and for cancer progression. The intralobular tissue supports the development of acini within TDLUs, while the interlobular tissue supports the function of ducts and myoepithelial cells.

Although murine models have extensively been used for studying human mammary gland development and tumorigenesis in vivo, a major limitation of these models is the anatomical and histological dissimilarities between mice and humans. The terminal end buds in mice which are functionally akin to TDLUs do not show as extensive branching as TDLUs and are histologically different. Most strikingly the stromal compartment of the mouse is mainly composed of adipose tissue with a limited amount of the collagenous stroma found abundantly in the human mammary tissue [4,5]. Nevertheless, mouse models have long served as great tools to advance our understanding of mammary gland biology.

## 2. The Lineage Relationship and Cellular Plasticity of the Breast Epithelium

From the earliest visible signs in the embryo, mammary gland development is dependent on crosstalk and signaling between the epidermis and the underlying stroma (reviewed in ([6,7,8,9,10,11,12]). Breast gland development begins as early as week 4 of gestation, with the appearance of a bilateral milk line, or mammary crest; a thickening of the epidermis that over the subsequent weeks is constricted to the future area of the breast gland in the thoracic region. Milk line specification is a highly choreographed interaction between the epidermis, dermal mesenchyme, and underlying somite signaling, with canonical, Wnt signaling cascade as an important aspect [11,12]. However, development in utero is limited to a rudimentary (albeit functional) ductal tree that invades the underlying mesenchyme, with the nipple and areolar tissue on the surface. Between birth and the onset of puberty, the mammary gland remains quiescent. In females, at the onset of puberty, drastic changes occur with respect to the mammary gland, which grows profusely, expanding into the surrounding stroma, that undergoes simultaneous remodeling. In addition to the global effects of growth hormone and IGF-1, which have been shown to be important in ductal growth and general mammary gland development [10,13], the onset of puberty also introduces increased levels of estrogen, progesterone, and prolactin. In particular, estrogen is important for producing the rapid growth of the mammary gland during puberty, in part via its induction of amphiregulin (AREG) expression, driving ductal elongation and cellular proliferation [14]. In addition, progesterone supports the differentiation of acini in TDLUs [15]. Other ligands of the EGFR family have been associated with having roles in the normal development of the breast gland, e.g., EGF, HGF, and TGFα [16,17]. These ligands have been shown to be expressed in the stroma, which is important to note here, as it has strong implications for breast cancer discussed later in this review. From the onset of puberty, cellular dynamics of the female breast epithelium follow the menstruation cycle where branching ducts expand and differentiate to prepare for pregnancy under the hormonal influence of ovarian-derived estrogen and progesterone [18,19]. Changes to the composition of the stroma have also been described, further demonstrating the dynamics in crosstalk between the stroma and epithelium [20]. If pregnancy does not occur, the TDLUs involute again, only to repeat the proliferative phase in the next menstruation cycle. If pregnancy occurs, the mammary gland reaches its fully differentiated state, with the TDLU acini producing milk after birth, that is pushed towards the nipples by myoepithelial cells upon suckling. Mammary gland development during pregnancy is driven by signals from the ovaries and placenta (estrogen and progesterone) and pituitary (prolactin), along with several paracrine factors such as members of the Notch pathway [21,22]. After birth, progesterone levels fall, which, in conjunction with the offspring breastfeeding, results in the commencement of lactation (galactogenesis) via increased prolactin signaling. After weaning, hormonal changes cause the fully differentiated mammary gland to involute again and become quiescent, apart from the menstrual cycle changes described earlier. Involution is driven by apoptosis of the epithelial cells and remodeling (softening) of the stroma of the breast gland, followed by an increase in adipocyte numbers. The initial process takes a few days to weeks, with the subsequent stroma remodeling taking considerably longer, with high variability between individuals, potentially influencing breast cancer risk [23,24].

During the previously discussed developmental phases of the breast gland, a certain dynamic potential is required from the epithelial cells. They must respond to several hormones, both systemic and local, and proliferate, migrate and involute in response. This makes mammary epithelial cells inherently predisposed to aberrant signaling changes that can occur during breast cancer initiation. Cells that accumulate mutations enhancing or preventing any of the aforementioned developmental processes can attain a premalignant phenotype. Obviously, not all epithelial mammary cells are the same. In addition to the terminally differentiated LEPs and MEPS, within the ductoalveolar tree there exist progenitor cells that drive the proliferation and remodeling of the gland. These cells are often referred to as stem cells, or basal cells, as they have not reached full differentiation and can repopulate the breast gland in repeated proliferative cycles [25]. The lineage relationship between the luminal and myoepithelial cells has been debated for decades. Due to the differences in accessibility, lineage dynamics have mostly been studied in mice [26], but with the advent of advanced sequencing methods such as single-cell RNA sequencing, and detailed screening using antibodies and biopsies, researchers have been able to discern the developmental tree representing the human breast gland [27,28,29,30]. In short, evidence indicates that within the breast gland are cells (basal cells) that can differentiate into either MEPs or luminal precursors, which then differentiate into alveolar or ductal LEPs. These precursor cells persist within the breast gland throughout the person’s lifetime. The Notch signaling pathway, central to a wide array of cellular differentiation processes throughout the body, is likewise a key player in mammary gland development and maintenance of mammary stem cells. Several studies have demonstrated the implication of the Notch pathway in self-renewal and differentiation of mammary stem cells, many in animal models (reviewed in [22]), and more recently in sophisticated three-dimensional models using human cells [31].

## 3. Breast Cancer

Due to the fetal development of the breast gland in both males and females, breast cancer (BC) can affect anyone; however, women are more susceptible to BC due to the hormonal-driven pubertal development described earlier. BC can be diagnosed at any age, but prevalence increases with age up to 70 years [32]. Tumors progress from normal tissue to carcinoma in situ, followed by invasion into the surrounding stroma, lymph nodes, and subsequently distant organs as metastatic disease. It is commonly recognized that breast tumors mainly arise from the luminal cell compartment. On the other hand, the myoepithelial cells are thought to be a terminally differentiated cell type surrounding the proliferative luminal epithelial cells. Indeed, the fully differentiated myoepithelial cells have been assigned the role of tumor suppressors as their presence is the hallmark of breast cancer in situ [33,34,35], whereas the absence of MEPs indicates invasive cancer. While referred to as a single term “Breast cancer”, it is anything but a single term. Rather, BC is an aggregate term describing a highly diverse and complex disease [36]. Throughout the progress of cancer initiation and progression, the cells of the tumor are under selective pressure for increased survival and proliferation—a classic Darwinian model of natural selection [37]. This has the implication that while there are similarities between tumors (to be discussed momentarily) no tumors are the same. Mutations may arise within populations of a developing tumor that provide enhanced proliferation and/or survival, so that even within a given tumor there exist several lineages of cells harboring different mutations. These subpopulations can therefore potentially respond differently to treatments [38]. Clinicians and researchers have for decades classified BC into sub-groups based on the presence (or absence) of several markers.

First, are the tumors expressing hormone receptors (HR), where a subset of cells (as low as 1%) within the tumor express either estrogen receptor alpha (ERα) or progesterone receptor (PR), or both. These tumors, which generally affect postmenopausal women in greater frequencies [39,40], are more numerous (up to 70%) than tumors with amplification of human epidermal growth factor-2 (HER2) or triple negative (TN) tumors, lacking both HR and HER2 expression [41]. The HR+ tumors in general have a lower grade than HER2 or TN tumors, with the cells in the tumor resembling normal breast cells to a greater extent, even though the tumor histoarchitecture is vastly different. HR+ tumors are suggested to arise from luminal progenitors and have acquired a mutation signature that differentiates them from basal-like tumors discussed later [42]. HR+ tumors are thought to be driven by hormonal signaling, and the presence of the hormone receptors means that these tumors can be targeted specifically with anti-hormone drugs, such as tamoxifen, which inhibits the function of the ERα, or aromatase inhibitors (AI) that inhibit the formation of estrogen in tissues [43]. The use of tamoxifen and AIs has greatly improved life expectancy in recent decades.

Second, are the tumors expressing high levels of the tyrosine kinase receptor HER2, usually due to a genetic amplification of the ErbB2 gene. These tumors are less frequent (up to 15%) but more aggressive than HR+ tumors and affect all age groups [39,40]. HER2 is a member of the EGFR receptor tyrosine kinase (RTK) family of four proteins. HER2 can heterodimerize with the other RTKs upon binding to a ligand leading to a downstream signaling cascade. Interestingly HER2 has no known ligand itself but is always in a so-called active conformation—meaning it is always available to dimerize, and if it is overexpressed, it can form homodimers that are ligand-independent—a classic hallmark of an oncogene. In tumors, HER2 expression is graded from 0, with no expression to +3 with high expression. If a tumor scores +3 or +2 with genetic amplification, it is classified as a HER2 tumor. HER2 tumors can be treated with trastuzumab, a monoclonal antibody that prevents HER2 dimerization. [43].

Third, are the so-called triple-negative (TN) tumors, named by the lack of any of the previously mentioned tumor markers. These are the rarest of the three tumor groups, but the most aggressive. TN tumors also affect premenopausal women in greater frequencies [44]. TN tumors often have high grade—meaning that cells within the tumor are poorly differentiated and dissimilar to normal breast cells. TN tumors share many characteristics with the progenitor population of the mammary gland [45], and research has suggested that many TN tumors have their origins from these cells. Mammary progenitor proliferation and survival is tightly controlled by the microenvironment; the stroma and surrounding epithelial cells. Many of these factors are still poorly understood, but dysregulation of Notch signaling has been linked to breast cancer initiation, where aberrant signaling results in changes to differentiation and proliferation, especially in the triple-negative breast cancer (TNBC) group [46]. Interestingly research has shown that pregnancy has a protective effect against breast cancer and that in particular lactation has a protective effect against TNBC, in part due to a depletion of this pool of precursor cells [47,48,49,50].

**Figure 2:**
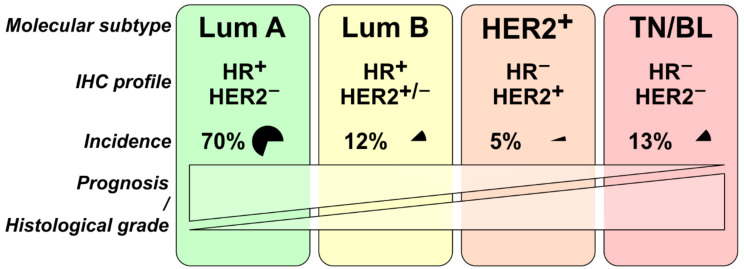
Schematic representation of selected breast tumor statistics, based on [55]. Lum = luminal, TN/BL = triple-negative/basal-like, HR = hormone receptor. Statistics adapted from [39,41], based on identifiable tumors.

In addition to histopathological subtypes, breast cancer is classified into molecular subtypes based on genetic signatures. The gene expression patterns of human breast tumors were first revealed in 2000 by Perou et al. [51] who used DNA microarray to define four subtypes of breast cancer, namely luminal, normal-like, HER2-enriched, and basal-like. However, later they divided the luminal subtype into luminal A and luminal B/C [52,53]. In 2009, Parker et al. reduced the gene list for profiling breast cancer subtypes to a gene set of 50 signature genes with the Prediction Analysis on Microarrays (PAM50) which currently is widely used for breast cancer subtyping. Herein, the heterogeneity of breast cancer is reflected by four main subtypes: luminal A, luminal B, HER2-enriched, and basal-like [54]. These molecular subtypes show differences with regard to incidence and prognosis, which in part overlap with histopathological subtypes. A summary of this chapter can be visualized in Figure 2.

## 4. Breast Cancer Initiation

The cellular origin of breast cancer has been debated for decades, but accumulating evidence is linked to mutations in stem or progenitor cells within the epithelial compartment (reviewed in [56]) and those mutations (depending on the type of mutation and context of the cell and its environment) can give rise to the various histopathological sub-types of breast cancer [37,38,57]. The basement membrane (BM), a specialized type of ECM, rich in collagen IV and laminin, separates the epithelial and the stromal compartment and provides a platform for the establishment of cellular polarity as well as providing physical and biochemical cues to the epithelial cells. The definition of breast cancer in situ is a tumor growth surrounded by a layer of myoepithelial cells and an intact basement membrane. Structural reorganization of the basement membrane is a prerequisite for cancer cell invasion and metastasis (reviewed in [58]). Invasive breast cancer has escaped through the layer of myoepithelial cells and BM and mixed with the surrounding stroma. This results in reciprocal interactions between cancer cells and the stroma. The process of invasion is not fully understood but two main schools exist, migration of single cells or collective migration of a group of cancer cells [59,60].

Another contributing factor to cancer initiation and subsequent progression is the immune response. There are many similarities between tumors and wounds, and the signaling pathways active within. Both lesions induce the recruitment of innate immune cells such as neutrophils that drive an inflammatory response, activating fibroblasts and promoting the formation of new blood vessels within the area [61,62]. In our paper from 2020, we demonstrate how aberrant signaling caused by the overexpression of HER2 induces the increased expression of Extracellular Matrix Protein 1 (ECM1). In that paper we showed that ECM1 led to the activation of endothelial cells, subsequently causing an increase in migration and invasion of epithelial cells [63]. ECM1 has been shown to be an important inflammatory response protein in other diseases such as inflammatory bowel disease through the activation of macrophages [64]. Macrophages have been shown to be clinically significant in the breast tumor environment, where they are associated with crown-like structures. Here, activated macrophages engulf adipocytes, and secrete pro-inflammatory molecules such as IL1-ß, IL-6, TNF-alpha, and VEGF, promoting proliferation and remodeling of the tumor tissue [65,66]. This inflammatory response and its importance in tumor initiation and progression is emphasized by the observed protective role of non-steroidal anti-inflammatory drugs such as aspirin and ibuprofen, making inflammation an important factor to consider when modeling cancer progression and cellular plasticity [67,68].

## 5. Epithelial Plasticity in Breast Cancer

One major contributing phenomenon to epithelial plasticity in the breast gland during cancer progression is epithelial to mesenchymal transition (EMT), and its reverse process mesenchymal to epithelial transition (MET), both important developmental processes that are hijacked in a number of cancer types including breast cancer [36]. It is generally accepted that epithelial cancer cells initially lose some epithelial properties and gain mesenchymal properties enabling their invasion into adjacent tissues and extravasation into the blood or lymphoid vessels. However complete EMT is rarely seen in cancer, rather partial EMT (pEMT) is by most researchers considered a frequently occurring event [69]. Here, epithelial cells lose polarization and several epithelial characteristics such as the expression of adhesion molecules such as E-cadherin, change shape to spindle/fibroblast-like phenotype, and gain several mesenchymal traits such as N-cadherin expression. The regulation of EMT is under the control of a number of transcription factors and non-coding RNAs [70,71,72,73]. It has long been widely accepted that EMT-like metastatic cancer cells needed to revert to the epithelial phenotype to be able to colonize distant tissues/organs, making the reverse process, MET, critical for the formation of distant metastasis. This is however debated, mainly due to a lack of convincing in vivo evidence and more recent data suggesting that increased malignancy and metastatic potential of breast cancer cells is correlated to the pEMT state rather than the complete epithelial or mesenchymal state [74,75,76]. Furthermore, the EMT-like phenotype has been linked to cancer stem cells (CSCs) and increased drug resistance [77,78]. Yet, as with malignancy and metastatic potential, drug resistance may depend on the degree of the pEMT state; however, this matter remains to be fully elucidated (reviewed in [79]).

We have previously shown that endothelial cells are able to induce EMT in breast epithelial cells [80]. Herein, we demonstrated that D492 a breast epithelial progenitor cell line that generates TDLU-like structures in 3D culture undergoes EMT when co-cultured with endothelial cells. A mesenchymal cell line derived from D492, referred to as D492M is non-tumorigenic, shows extensive EMT phenotype, and has lost epithelial plasticity [80]. In a subsequent study, we showed that microRNA-200c-141 and p63 were the necessary factors to induce MET in the D492M cell line [70]. Moreover, we have generated an oncogenic version of D492 by overexpressing the HER2 oncogene. D492HER2 is highly tumorigenic, displays mixed epithelial/mesenchymal phenotype, and shows more epithelial plasticity than D492M [81].

## 6. The Stromal Microenvironment Dominantly Affects Breast Morphogenesis and Cancer Progression

The stroma is composed of an extracellular matrix, entrapped growth factors, and resident cell types such as fibroblasts, endothelial cells, immune cells, and adipocytes. It is becoming more and more clear that stroma plays an instrumental part in breast morphogenesis and cancer progression. Fibroblasts are the most prominent cell type in the stroma and their role in tissue morphogenesis and cancer progression has been widely recognized [82]. Cancer-associated fibroblasts (CAF) also known as myofibroblasts are the dominant creators of the tumor microenvironment that facilitate further tumor progression [83].

Fibroblasts, recently shown to differ between the ductal and TDLU area [3], are the main producers of components of the extracellular matrix (ECM) such as collagen 1, fibronectin, and elastin. As such, the fibroblasts regulate the stiffness of the ECM which has a large effect on the epithelial phenotype under normal and cancerous conditions. In that regard, increased mammographic density is positively correlated with increased risk for breast cancer [84]. Moreover, multiple studies have demonstrated that activated fibroblasts have a profound influence on breast cancer initiation and progression [85,86]. Indeed, solid tumors are to a large extent composed of cancer cells and fibrotic tissue, and tumors have been referred to as “wounds that never heal”. CAF contributes to increased stiffness of ECM and the altered microenvironment has been shown to induce mechanoreciprocity that promotes further cancer invasion [87,88].

The growth of solid tumors generates hypoxic conditions that induce both cancer cells and stromal cells to initiate a pro-angiogenic program that attracts surrounding microvessels to sprout into the tumor mass to deliver and remove O_2_ and nutrients and CO_2_ and waste products, respectively. Previously, endothelial cells (ECs) were viewed as an inert passive cell type coating the capillary network. We and others have shown that ECs have proliferative and morphogenic effects on normal and cancerous breast epithelial cells [80,89]. Furthermore, we have shown that ECs induce EMT in breast progenitor cell lines and cancer cells [80]. Thus, ECs may act as an important player in breast morphogenesis as well as being an active participant in the tumor microenvironment promoting cancer progression.

## 7. Concluding Remarks

Cellular plasticity is a developmental process that allows cells to change phenotype as a response to intrinsic and extrinsic signals. This is very important during embryonic development as temporal and spatial cellular changes arrange organ development to a large extent. This is also important in some adult organs such as the female mammary gland, which undergoes substantial remodeling from the onset of menarche until menopause and more drastically during pregnancy. Epithelial stem and progenitor cells are responsible for the remodeling phase in the female breast and the origin of breast cancer has been linked to the presence of these cells. Indeed, the term “cancer stem cells” has been used due to the similarities between the properties of stem cells and cancer cells. These properties include cellular plasticities such as partial EMT, longevity, and increased resistance to apoptosis. Solid cancers such as breast cancer are not only composed of cancer cells but also a cellular-rich microenvironment/stroma. Dynamic reciprocal interactions exist between cancer cells and stroma and cancer cells induce functional and structural changes in stroma such as induction of myofibroblasts which respond with increased expression of multiple factors that promote cancer invasion. In summary, a better understanding of the heterotypic interactions between breast cancer cells and the surrounding microenvironment including the cellular plasticity of cancer cells is of great importance as this may predict the aggressiveness of invasive tumors and provide a platform for new treatment options. In that regard, an increased understanding of stem cell biology in the normal and neoplastic breast gland will advance this research as stem or progenitor cells may be the cell type of origin for a number of breast cancers.

## References

[1] Yousef H., Alhajj M., Sharma S. (2021). Anatomy, Skin (Integument), Epidermis. StatPearls.

[2] Morsing M., Kim J., Villadsen R., Goldhammer N., Jafari A., Kassem M., Petersen O.W., Rønnov-Jessen L. (2020). Fibroblasts direct differentiation of human breast epithelial progenitors. Breast Cancer Res..

[3] Morsing M., Klitgaard M.C., Jafari A., Villadsen R., Kassem M., Petersen O.W., Rønnov-Jessen L. (2016). Evidence of two distinct functionally specialized fibroblast lineages in breast stroma. Breast Cancer Res..

[4] Dontu G., Ince T.A. (2015). Of mice and women: A comparative tissue biology perspective of breast stem cells and differentiation. J. Mammary Gland. Biol. Neoplasia.

[5] McNally S., Stein T. (2017). Overview of Mammary Gland Development: A Comparison of Mouse and Human. Methods Mol. Biol..

[6] Musumeci G., Castrogiovanni P., Szychlinska M.A., Aiello F.C., Vecchio G.M., Salvatorelli L., Magro G., Imbesi R. (2015). Mammary gland: From embryogenesis to adult life. Acta Histochem..

[7] Lteif A., Javed A. (2013). Development of the Human Breast. Semin. Plast. Surg..

[8] Robinson G.W., Karpf A.B.C., Kratochwil K. (1999). Regulation of Mammary Gland Development by Tissue Interaction. J. Mammary Gland. Biol. Neoplasia.

[9] Spina E., Cowin P. (2021). Embryonic mammary gland development. Semin. Cell Dev. Biol..

[10] Macias H., Hinck L. (2012). Mammary gland development. Wiley Interdiscip. Rev. Dev. Biol..

[11] Cowin P., Wysolmerski J. (2010). Molecular Mechanisms Guiding Embryonic Mammary Gland Development. Cold Spring Harb. Perspect. Biol..

[12] Boras-Granic K., Chang H., Grosschedl R., Hamel P.A. (2006). Lef1 is required for the transition of Wnt signaling from mesenchymal to epithelial cells in the mouse embryonic mammary gland. Dev. Biol..

[13] Ruan W., Kleinberg D.L. (1999). Insulin-Like Growth Factor I Is Essential for Terminal End Bud Formation and Ductal Morphogenesis during Mammary Development1. Endocrinology.

[14] Ciarloni L., Mallepell S., Brisken C. (2007). Amphiregulin is an essential mediator of estrogen receptor α function in mammary gland development. Proc. Natl. Acad. Sci. USA.

[15] Lamb C.A., Fabris V.T., Lanari C. (2020). Progesterone and breast. Best Pract. Res. Clin. Obstet. Gynaecol..

[16] Haslam S.Z., Woodward T.L. (2003). Host microenvironment in breast cancer development: Epithelial-cell–stromal-cell interactions and steroid hormone action in normal and cancerous mammary gland. Breast Cancer Res..

[17] Zhang H.-Z., Bennett J.M., Smith K.T., Sunil N., Haslam S.Z. (2002). Estrogen Mediates Mammary Epithelial Cell Proliferation in Serum-Free Culture Indirectly via Mammary Stroma-Derived Hepatocyte Growth Factor. Endocrinology.

[18] Ramakrishnan R., Khan S.A., Badve S. (2002). Morphological Changes in Breast Tissue with Menstrual Cycle. Mod. Pathol..

[19] Atashgaran V., Wrin J., Barry S.C., Dasari P., Ingman W.V. (2016). Dissecting the Biology of Menstrual Cycle-Associated Breast Cancer Risk. Front. Oncol..

[20] Ferguson J.E., Schor A.M., Howell A., Ferguson M.W.J. (1992). Changes in the extracellular matrix of the normal human breast during the menstrual cycle. Cell Tissue Res..

[21] Lamote I., Meyer E., Massart-Leën A.M., Burvenich C. (2004). Sex steroids and growth factors in the regulation of mammary gland proliferation, differentiation, and involution. Steroids.

[22] Chen W., Wei W., Yu L., Ye Z., Huang F., Zhang L., Hu S., Cai C. (2021). Mammary Development and Breast Cancer: A Notch Perspective. J. Mammary Gland. Biol. Neoplasia.

[23] Maskarinec G., Ju D., Horio D., Loo L.W.M., Hernandez B.Y. (2016). Involution of breast tissue and mammographic density. Breast Cancer Res..

[24] Jindal S., Narasimhan J., Borges V.F., Schedin P. (2020). Characterization of weaning-induced breast involution in women: Implications for young women’s breast cancer. NPJ Breast Cancer.

[25] Visvader J.E., Stingl J. (2014). Mammary stem cells and the differentiation hierarchy: Current status and perspectives. Genes Dev..

[26] Rios A.C., Fu N.Y., Lindeman G.J., Visvader J.E. (2014). In situ identification of bipotent stem cells in the mammary gland. Nature.

[27] Nguyen Q.H., Pervolarakis N., Blake K., Ma D., Davis R.T., James N., Phung A.T., Willey E., Kumar R., Jabart E. (2018). Profiling human breast epithelial cells using single cell RNA sequencing identifies cell diversity. Nat. Commun..

[28] Fridriksdottir A.J., Villadsen R., Morsing M., Klitgaard M.C., Kim J., Petersen O.W., Rønnov-Jessen L. (2017). Proof of region-specific multipotent progenitors in human breast epithelia. Proc. Natl. Acad. Sci. USA.

[29] Villadsen R., Fridriksdottir A.J., Rønnov-Jessen L., Gudjonsson T., Rank F., LaBarge M.A., Bissell M.J., Petersen O.W. (2007). Evidence for a stem cell hierarchy in the adult human breast. J. Cell Biol..

[30] Gray G.K., Li C.M.-C., Rosenbluth J.M., Selfors L.M., Girnius N., Lin J.-R., Schackmann R.C.J., Goh W.L., Moore K., Shapiro H.K. (2022). A human breast atlas integrating single-cell proteomics and transcriptomics. Dev. Cell.

[31] Rauner G., Jin D.X., Miller D.H., Gierahn T.M., Li C.M., Sokol E.S., Feng Y.-X., Mathis R.A., Love J.C., Gupta P.B. (2021). Breast tissue regeneration is driven by cell-matrix interactions coordinating multi-lineage stem cell differentiation through DDR1. Nat. Commun..

[32] Surveillance Research Program, N.C.I. (2021). SEER*Explorer: An. Interactive Website for SEER Cancer Statistics [Internet].

[33] Pandey R., Saidou J., Watabe K. (2010). Role of myoepithelial cells in breast tumor progression. Front. Biosci..

[34] Adriance M.C., Inman J.L., Petersen O.W., Bissell M.J. (2005). Myoepithelial cells: Good fences make good neighbors. Breast Cancer Res..

[35] Sternlicht M.D., Kedeshian P., Shao Z.M., Safarians S., Barsky S.H. (1997). The human myoepithelial cell is a natural tumor suppressor. Clin. Cancer Res..

[36] Loibl S., Poortmans P., Morrow M., Denkert C., Curigliano G. (2021). Breast cancer. Lancet.

[37] Fortunato A., Boddy A., Mallo D., Aktipis A., Maley C.C., Pepper J.W. (2017). Natural Selection in Cancer Biology: From Molecular Snowflakes to Trait Hallmarks. Cold Spring Harb. Perspect. Med..

[38] Vitale I., Shema E., Loi S., Galluzzi L. (2021). Intratumoral heterogeneity in cancer progression and response to immunotherapy. Nat. Med..

[39] Howlader N., Altekruse S.F., Li C.I., Chen V.W., Clarke C.A., Ries L.A.G., Cronin K.A. (2014). US Incidence of Breast Cancer Subtypes Defined by Joint Hormone Receptor and HER2 Status. JNCI J. Natl. Cancer Inst..

[40] Łukasiewicz S., Czeczelewski M., Forma A., Baj J., Sitarz R., Stanisławek A. (2021). Breast Cancer-Epidemiology, Risk Factors, Classification, Prognostic Markers, and Current Treatment Strategies-An Updated Review. Cancers.

[41] Nasim Z., Girtain C., Gupta V., Patel I., Hossain M.A. (2020). Breast Cancer Incidence and Behavior in Younger Patients: A Study From the Surveillance, Epidemiology and End Results Database. World J. Oncol..

[42] Zhou J., Chen Q., Zou Y., Chen H., Qi L., Chen Y. (2019). Stem Cells and Cellular Origins of Breast Cancer: Updates in the Rationale, Controversies, and Therapeutic Implications. Front. Oncol..

[43] Kay C., Martínez-Pérez C., Meehan J., Gray M., Webber V., Dixon J.M., Turnbull A.K. (2021). Current trends in the treatment of HR+/HER2+ breast cancer. Future Oncol..

[44] Yin L., Duan J.-J., Bian X.-W., Yu S.-C. (2020). Triple-negative breast cancer molecular subtyping and treatment progress. Breast Cancer Res..

[45] Prat A., Parker J.S., Karginova O., Fan C., Livasy C., Herschkowitz J.I., He X., Perou C.M. (2010). Phenotypic and molecular characterization of the claudin-low intrinsic subtype of breast cancer. Breast Cancer Res..

[46] Giuli M.V., Giuliani E., Screpanti I., Bellavia D., Checquolo S. (2019). Notch Signaling Activation as a Hallmark for Triple-Negative Breast Cancer Subtype. J. Oncol..

[47] Siwko S.K., Dong J., Lewis M.T., Liu H., Hilsenbeck S.G., Li Y. (2008). Evidence that an early pregnancy causes a persistent decrease in the number of functional mammary epithelial stem cells—Implications for pregnancy-induced protection against breast cancer. Stem Cells.

[48] Russo J., Moral R., Balogh G.A., Mailo D., Russo I.H. (2005). The protective role of pregnancy in breast cancer. Breast Cancer Res..

[49] Redondo C.M., Gago-Domínguez M., Ponte S.M., Castelo M.E., Jiang X., García A.A., Fernández M.P., Tomé M.A., Fraga M., Gude F. (2012). Breast feeding, parity and breast cancer subtypes in a Spanish cohort. PLoS ONE.

[50] Fortner R.T., Sisti J., Chai B., Collins L.C., Rosner B., Hankinson S.E., Tamimi R.M., Eliassen A.H. (2019). Parity, breastfeeding, and breast cancer risk by hormone receptor status and molecular phenotype: Results from the Nurses’ Health Studies. Breast Cancer Res..

[51] Perou C.M., Sørlie T., Eisen M.B., van de Rijn M., Jeffrey S.S., Rees C.A., Pollack J.R., Ross D.T., Johnsen H., Akslen L.A. (2000). Molecular portraits of human breast tumours. Nature.

[52] Sørlie T., Perou C.M., Tibshirani R., Aas T., Geisler S., Johnsen H., Hastie T., Eisen M.B., van de Rijn M., Jeffrey S.S. (2001). Gene expression patterns of breast carcinomas distinguish tumor subclasses with clinical implications. Proc. Natl. Acad. Sci. USA.

[53] Sorlie T., Tibshirani R., Parker J., Hastie T., Marron J.S., Nobel A., Deng S., Johnsen H., Pesich R., Geisler S. (2003). Repeated observation of breast tumor subtypes in independent gene expression data sets. Proc. Natl. Acad. Sci. USA.

[54] Parker J.S., Mullins M., Cheang M.C., Leung S., Voduc D., Vickery T., Davies S., Fauron C., He X., Hu Z. (2009). Supervised risk predictor of breast cancer based on intrinsic subtypes. J. Clin. Oncol..

[55] Sims A.H., Howell A., Howell S.J., Clarke R.B. (2007). Origins of breast cancer subtypes and therapeutic implications. Nat. Clin. Pract. Oncol..

[56] Visvader J.E. (2009). Keeping abreast of the mammary epithelial hierarchy and breast tumorigenesis. Genes Dev..

[57] Zhang M., Lee A.V., Rosen J.M. (2017). The Cellular Origin and Evolution of Breast Cancer. Cold Spring Harb. Perspect. Med..

[58] Winkler J., Abisoye-Ogunniyan A., Metcalf K.J., Werb Z. (2020). Concepts of extracellular matrix remodelling in tumour progression and metastasis. Nat. Commun..

[59] Mayor R., Etienne-Manneville S. (2016). The front and rear of collective cell migration. Nat. Rev. Mol. Cell Biol..

[60] Lintz M., Muñoz A., Reinhart-King C.A. (2017). The Mechanics of Single Cell and Collective Migration of Tumor Cells. J. Biomech. Eng..

[61] de Visser K.E., Coussens L.M. (2006). The inflammatory tumor microenvironment and its impact on cancer development. Contrib. Microbiol..

[62] Dvorak H.F. (2015). Tumors: Wounds that do not heal-redux. Cancer Immunol. Res..

[63] Steinhaeuser S.S., Morera E., Budkova Z., Schepsky A., Wang Q., Rolfsson O., Riedel A., Krueger A., Hilmarsdottir B., Maelandsmo G.M. (2020). ECM1 secreted by HER2-overexpressing breast cancer cells promotes formation of a vascular niche accelerating cancer cell migration and invasion. Lab. Invest..

[64] Zhang Y., Li X., Luo Z., Ma L., Zhu S., Wang Z., Wen J., Cheng S., Gu W., Lian Q. (2020). ECM1 is an essential factor for the determination of M1 macrophage polarization in IBD in response to LPS stimulation. Proc. Natl. Acad. Sci. USA.

[65] Iyengar N.M., Hudis C.A., Dannenberg A.J. (2013). Obesity and inflammation: New insights into breast cancer development and progression. Am. Soc. Clin. Oncol. Educ. Book.

[66] Faria S.S., Corrêa L.H., Heyn G.S., de Sant’Ana L.P., Almeida R.D.N., Magalhães K.G. (2020). Obesity and Breast Cancer: The Role of Crown-Like Structures in Breast Adipose Tissue in Tumor Progression, Prognosis, and Therapy. J. Breast Cancer.

[67] Harris R.E., Beebe-Donk J., Doss H., Burr Doss D. (2005). Aspirin, ibuprofen, and other non-steroidal anti-inflammatory drugs in cancer prevention: A critical review of non-selective COX-2 blockade (review). Oncol. Rep..

[68] Moris D., Kontos M., Spartalis E., Fentiman I.S. (2016). The Role of NSAIDs in Breast Cancer Prevention and Relapse: Current Evidence and Future Perspectives. Breast Care.

[69] Yang J., Antin P., Berx G., Blanpain C., Brabletz T., Bronner M., Campbell K., Cano A., Casanova J., Christofori G. (2020). Guidelines and definitions for research on epithelial–mesenchymal transition. Nat. Rev. Mol. Cell Biol..

[70] Hilmarsdóttir B., Briem E., Sigurdsson V., Franzdóttir S.R., Ringnér M., Arason A.J., Bergthorsson J.T., Magnusson M.K., Gudjonsson T. (2015). MicroRNA-200c-141 and ∆Np63 are required for breast epithelial differentiation and branching morphogenesis. Dev. Biol..

[71] Budkova Z., Sigurdardottir A.K., Briem E., Bergthorsson J.T., Sigurdsson S., Magnusson M.K., Traustadottir G.A., Gudjonsson T., Hilmarsdottir B. (2020). Expression of ncRNAs on the DLK1-DIO3 Locus Is Associated With Basal and Mesenchymal Phenotype in Breast Epithelial Progenitor Cells. Front. Cell Dev. Biol..

[72] Dvinge H., Git A., Gräf S., Salmon-Divon M., Curtis C., Sottoriva A., Zhao Y., Hirst M., Armisen J., Miska E.A. (2013). The shaping and functional consequences of the microRNA landscape in breast cancer. Nature.

[73] Zhang B., Pan X., Cobb G.P., Anderson T.A. (2007). microRNAs as oncogenes and tumor suppressors. Dev. Biol.

[74] Williams E.D., Gao D., Redfern A., Thompson E.W. (2019). Controversies around epithelial-mesenchymal plasticity in cancer metastasis. Nat. Rev. Cancer.

[75] Gupta P.B., Pastushenko I., Skibinski A., Blanpain C., Kuperwasser C. (2019). Phenotypic Plasticity: Driver of Cancer Initiation, Progression, and Therapy Resistance. Cell Stem Cell.

[76] Pastushenko I., Brisebarre A., Sifrim A., Fioramonti M., Revenco T., Boumahdi S., Van Keymeulen A., Brown D., Moers V., Lemaire S. (2018). Identification of the tumour transition states occurring during EMT. Nature.

[77] Derynck R., Weinberg R.A. (2019). EMT and Cancer: More Than Meets the Eye. Dev. Cell.

[78] Liu S., Cong Y., Wang D., Sun Y., Deng L., Liu Y., Martin-Trevino R., Shang L., McDermott S.P., Landis M.D. (2014). Breast cancer stem cells transition between epithelial and mesenchymal states reflective of their normal counterparts. Stem Cell Rep..

[79] De Las Rivas J., Brozovic A., Izraely S., Casas-Pais A., Witz I.P., Figueroa A. (2021). Cancer drug resistance induced by EMT: Novel therapeutic strategies. Arch. Toxicol..

[80] Sigurdsson V., Hilmarsdottir B., Sigmundsdottir H., Fridriksdottir A.J., Ringnér M., Villadsen R., Borg A., Agnarsson B.A., Petersen O.W., Magnusson M.K. (2011). Endothelial induced EMT in breast epithelial cells with stem cell properties. PLoS ONE.

[81] Ingthorsson S., Andersen K., Hilmarsdottir B., Maelandsmo G.M., Magnusson M.K., Gudjonsson T. (2016). HER2 induced EMT and tumorigenicity in breast epithelial progenitor cells is inhibited by coexpression of EGFR. Oncogene.

[82] Houthuijzen J.M., Jonkers J. (2018). Cancer-associated fibroblasts as key regulators of the breast cancer tumor microenvironment. Cancer Metastasis Rev..

[83] Piersma B., Hayward M.K., Weaver V.M. (2020). Fibrosis and cancer: A strained relationship. Biochim. Et Biophys. Acta Rev. Cancer.

[84] Mokhtary A., Karakatsanis A., Valachis A. (2021). Mammographic Density Changes over Time and Breast Cancer Risk: A Systematic Review and Meta-Analysis. Cancers.

[85] Ping Q., Yan R., Cheng X., Wang W., Zhong Y., Hou Z., Shi Y., Wang C., Li R. (2021). Cancer-associated fibroblasts: Overview, progress, challenges, and directions. Cancer Gene Ther..

[86] Rønnov-Jessen L., Petersen O.W., Koteliansky V.E., Bissell M.J. (1995). The origin of the myofibroblasts in breast cancer. Recapitulation of tumor environment in culture unravels diversity and implicates converted fibroblasts and recruited smooth muscle cells. J. Clin. Invest..

[87] Maller O., Drain A.P., Barrett A.S., Borgquist S., Ruffell B., Zakharevich I., Pham T.T., Gruosso T., Kuasne H., Lakins J.N. (2021). Tumour-associated macrophages drive stromal cell-dependent collagen crosslinking and stiffening to promote breast cancer aggression. Nat. Mater..

[88] Northey J.J., Barrett A.S., Acerbi I., Hayward M.K., Talamantes S., Dean I.S., Mouw J.K., Ponik S.M., Lakins J.N., Huang P.J. (2020). Stiff stroma increases breast cancer risk by inducing the oncogene ZNF217. J. Clin. Investig..

[89] Ingthorsson S., Sigurdsson V., Fridriksdottir A.J.R., Jonasson J.G., Kjartansson J., Magnusson M.K., Gudjonsson T. (2010). Endothelial cells stimulate growth of normal and cancerous breast epithelial cells in 3D culture. BMC Res. Notes.

